# Pandemic Fear and Literature: Observations from Jack London’s The Scarlet Plague

**DOI:** 10.3201/eid2010.130278

**Published:** 2014-10

**Authors:** Michele Augusto Riva, Marta Benedetti, Giancarlo Cesana

**Affiliations:** University of Milano Bicocca, Monza, Italy (M.A. Riva, G. Cesana);; Università Cattolica del Sacro Cuore, Milan, Italy (M. Benedetti)

**Keywords:** Plague, history, medicine in literature, pandemic, bacteria, Jack London, The Scarlet Plague

The Scarlet Plague, originally published by Jack London in 1912, was one of the first examples of a postapocalyptic fiction novel in modern literature (*1*). Set in a ravaged and wild America, the story takes place in 2073, sixty years after the spread of the Red Death, an uncontrollable epidemic that depopulated and nearly destroyed the world in 2013. One of the few survivors, James Howard Smith, alias “Granser,” tells his incredulous and near-savage grandsons how the pandemic spread in the world and about the reactions of the people to contagion and death. Even though it was published more than a century ago, The Scarlet Plague feels contemporary because it allows modern readers to reflect on the worldwide fear of pandemics, a fear that remains very much alive.

By exploring the motif of the plague, a consistent and well-spread topos (i.e., theme) in literature (*2**–**4*), London’s novel is part of a long literary tradition, inviting the reader to reflect on the ancestral fear of humans toward infectious diseases. In the ancient world, plague and pestilence were rather frequent calamities, and ordinary people were likely to have witnessed or heard vivid and scary reports about their terrible ravages (*5*). When plague spread, no medicine could help, and no one could stop it from striking; the only way to escape was to avoid contact with infected persons and contaminated objects (*6*). The immense fright was also fueled by a belief in the supernatural origin of pandemics, which were often believed to be provoked by offenses against divinities. In the Bible (e.g., Exodus 9:14, Numbers 11:33, 1 Samuel 4:8, Psalms 89:23, Isaiah 9:13), the plague was viewed as one of God’s punishments for sins, so the frightening description of its spread was interpreted as a warning to the Israelites to behave morally. This causal relationship between plague and sin is seen also in Greek literary texts, such as Homer’s Iliad and Sophocles’ Oedipus the King (429 BCE).

In contrast, the Greek historian Thucydides (c. 460–395 BCE), in his History of the Peloponnesian War, and the Latin poet Lucretius (c. 99–55 BCE), in his De Rerum Natura, refuted a supernatural origin of the disease and focused their descriptions on the uncontrolled fear of contagion among the public. According to these authors, plague did not discriminate between the good and the evil but brought about the loss of all social conventions and a rise in selfishness and avarice. 

Later medieval writings, such as The Decameron by Giovanni Boccaccio (1313–1375) and The Canterbury Tales by Geoffrey Chaucer (1343–1400), emphasized human behavior: the fear of contagion increased vices such as avarice, greed, and corruption, which paradoxically led to infection and thus to both moral and physical death (*7**,**8*). Human reactions to the plague are also the central themes of historical titles such as A Journal of the Plague Year by Daniel Defoe (1659–1731), a long, detailed narrative of events, anecdotes, and statistics regarding the Great Plague of London of 1665. In a similar manner, The Betrothed and History of the Column of Infamy, both written by Italian novelist Alessandro Manzoni (1785–1873), were extraordinary descriptions of the plague that struck Milan around 1630 (*9*). 

In English-language literature, The Last Man (1826) by English novelist Mary Shelley (1797–1851) was one of the first apocalyptic novels, telling of a future world that had been ravaged by a plague; a few persons appear to be immune and avoid contact with others. The concept of immunization in this book demonstrates that the author, most famous for the novel Frankenstein, had a deep understanding of contemporaneous theories about the nature of contagion. In 1842, the American poet and novelist Edgar Allan Poe (1809–1849) published The Masque of the Red Death, a short story unique in the literary tradition of the plague by focusing only on the metaphorical element of the topos. Through the personification of the plague, represented by a mysterious figure disguised as a Red Death victim, the author meditates on the inevitability of death; the issue is not that people die from the plague, but that people are plagued by death (*9*).

## The Scarlet Plague and the Fear of Pandemic

Jack London (1876–1916) was a US writer and journalist and author of classic novels including The Call of the Wild (1903) and White Fang (1906). He was also an active member of the Socialist Party of America, and his works often contained explicit critiques against capitalism and war. Numerous stories London wrote would today be classified as science fiction, and some had pandemics and infectious diseases as subjects. The Unparalleled Invasion (1910) described a biological warfare campaign launched from the United States and the other Western countries to arrest the uncontrolled growth of China’s population and protect European colonies in Asia from Chinese immigration. In The Scarlet Plague, London investigated many traditional issues of the literary topos of plague, ranging from a reflection on morality and justice to the contagion and clinical features of the disease. In particular, the author focused his attention on behavioral responses to a pandemic, showing the emergence of fear, irrationality, and selfishness in a previously civilized and modern society. This novel differed greatly from earlier writings related to plague because it reflected deeply the contemporary scientific discoveries on pathogens fostered by scientists such as Louis Pasteur (1822–1895) and Robert Koch (1843–1910). By the early 20th century, epidemics were no longer considered divine punishments or supernatural events; 19th century bacteriologists had demonstrated that they are caused by germs that infect humans, and epidemiologists and public health experts had shed light on the mechanisms of disease transmission, including suggestions of general preventive measures to limit pandemics (*10*). Despite these scientific developments, however, in London’s time, the general public’s fear of the invisible world of microorganisms was still high.

In the novel, at the beginning of the epidemic of Scarlet Death, the people appeared not to be alarmed because they “were sure that the bacteriologists would find a way to overcome this new germ, just as they had overcome other germs in the past” (*1*). Public trust in science was high in the 21st century society described by London. However, the people were soon frightened by “the astonishing quickness with which this germ destroyed human beings, and [by] the fact that it inevitably killed any human body it entered. … From the moment of the first signs of it, a man would be dead in an hour. Some lasted for several hours. Many died within ten or fifteen minutes of the appearance of the first signs” (*1*). Through details of the course of the illness, London made the plague more realistic and even more frightening:

“The heart began to beat faster and the heat of the body to increase. Then came the scarlet rash, spreading like wildfire over the face and body. Most persons never noticed the increase in heat and heart-beat, and the first they knew was when the scarlet rash came out. Usually, they had convulsions at the time of the appearance of the rash. But these convulsions did not last long and were not very severe. … The heels became numb first, then the legs, and hips, and when the numbness reached as high as his heart he died.” (*1*)

London wrote of the rapid decomposition of corpses, which immediately released billions of germs, accelerating the spread of the disease and causing problems for the scientists who were not able to quickly find a specific treatment. By the time a serum against the plague was discovered, it was too late to stop the epidemic. Medicine and scientific progress were defeated by plague, as testified by the heroic death of bacteriologists who “were killed in their laboratories even as they studied the germ of the Scarlet Death. … As fast as they perished, others stepped forth and took their places” (*1*).

The defeat of the science and medicine in which the people had placed trust generated fear in the population. London gave detailed insight into the human reactions to the spread of the disease. In particular, Granser tells his grandsons how the people started to run away from the cities in a blind panic:

“Thursday night the panic outrush for the country began. Imagine, my grandsons, people, thicker than the salmon-run you have seen on the Sacramento river, pouring out of the cities by millions, madly over the country, in vain attempt to escape the ubiquitous death. You see, they carried the germs with them. Even the airships of the rich, fleeing for mountain and desert fastnesses, carried the germs.” (*1*)

Yet there was no escape. Germs were spreading, fast and uncontrolled. Nothing could stop it, and the world was in a state of sheer panic never experienced before. People started behave unreasonably: “we did not act in this way when ordinary diseases smote us. We were always calm over such things, and sent for the doctors and nurses who knew just what to do” (*1*). The population reacted to the outbreak of the plague in 2 ways: most tried in vain to isolate themselves and fled to avoid the contagion, whereas a minority, mainly rioters, begun drinking, robbing, and sometimes even killing:

“In the midst of our civilization, down in our slums and labor-ghettos, we had bred a race of barbarians, of savages; and now, in the time of our calamity, they turned upon us like the wild beasts they were and destroyed us. And they destroyed themselves as well.” (*1*)

After the plague, civilization fell apart, and the few survivors, scattered in a primitive world, had to fight for survival, echoing Darwinian theories: “Civilization was crumbling, and it was each for himself” (*1*). As had some earlier writers, London raised a harsh critique against the society that is seen as the ultimate cause of the world’s destruction. In particular, in London’s opinion, capitalism led to the rise in population and to overcrowding, and overcrowding led to plague. Consequently, capitalism is presented as the ultimate cause of the pandemic and thus harshly criticized.

As the human race in London’s world was dying, the earth was being devastated by fires and conflagrations: “The smoke of the burning filled the heavens, so that the midday was as a gloomy twilight, and, in the shifts of wind, sometimes the sun shone through dimly, a dull red orb. Truly, my grandsons, it was like the last days of the end of the world” (*1*). The end of the world: this is how the pandemic was perceived. Not only did the people fear their own death but they also had the terrible feeling of being at the end of the world: the cities were being destroyed by fire; the people were fleeing away in hysteria. This immense panic grew even more, frightening and unprecedented because of the stop in communication with the rest of the world, a hopeless sign of death: “It was amazing, astounding, this loss of communication with the world. It was exactly as if the world had ceased, been blotted out” (*1*).

The brutality of the plague London presents is greater than that presented in previous works. The apocalyptic scenario illustrates a common fear of epidemics. In London’s novel—as today—scientists were aware of the risk of uncontrolled pandemics. London’s novel foresaw the first and most severe influenza pandemic in history, the Spanish influenza of 1918–1920, which began its spread only 6 years after the publication of The Scarlet Plague and caused the death of 20 million persons worldwide. In the novel, as in reality, human reactions to plague can vary greatly, but still all share a terrible fear, the fear of death—both as the end of one’s life and as the end of civilization.

## Conclusions

As London shows in his novel, pandemics can bring forth deeply rooted fears and modify human behavior greatly. The American novelist used the plague topos to criticize contemporary social structure: the destruction that follows the plague is both to be welcomed and despised. Indeed, the pandemic breaks the class barriers, but it also leads to the ruin of civilization. According to London’s socialist values, only human brotherhood enables society to survive. Despite the political views of the author, the pandemic issue would have appealed to London’s readers; in 1912, the American audience had recently experienced the San Francisco plague of 1900–1904, an epidemic of bubonic plague centered on San Francisco’s Chinatown (*11*). During this epidemic, the initial denial and obstructionism of authorities in California, who wanted to prevent the loss of revenue from trade stopped by quarantine, were highly criticized by media and public opinion (*11*). Curiously, only 1 year before the publication of The Scarlet Plague, American writer and muckraker Samuel Hopkins Adams (1871–1958) wrote an editorial, Public Health and Public Hysteria, in the first volume of the Journal of the American Public Health Association (*12*). In his article, Adams argued that public health awareness is generated and sustained when fear of disease induces hysteria in population; consequently, at that time leprosy, cholera, and scarlet fever were considered the major public health priorities, rather than other, more common diseases, such as measles, whooping cough, and tuberculosis (*12*).

Today, despite the development of antimicrobial drugs, infectious diseases and germs continue to generate fear, as recently demonstrated by the worldwide epidemics of influenza A(H1N1) in 2009, avian influenza A(H5N1) in 2005–2006, and severe acute respiratory syndrome (SARS) in 2003, as well as the potential for attacks with bioterrorism agents such as anthrax or smallpox (*13*). Several studies have been conducted to analyze and hypothesize about the emotional, cognitive, and behavioral responses to epidemics among the public, in particular to provide policy makers and emergency responders with information about public perception and behavior in the aftermath of biological disasters, such as a deadly epidemic (*13**,**14*). A recent study in Switzerland analyzed the lay perceptions of collectives implicated in the 2009 influenza A(H1N1) outbreak and found that physicians and researchers were considered “heroes” of the pandemic (*15*). As in London’s times, the study illustrated that the public placed trust mainly in scientists rather than in political authorities and states, which were thought to be partly ineffective (*15*). On the other hand, media and private corporations (e.g., the pharmaceutical industry), which are believed to take advantage of the spread of diseases and to create alarmism, are accused of being social “villains” (*15*), much as London criticized capitalists. However, recent outbreaks have demonstrated that even the scientific community may make mistakes in managing infectious disease (*16**,**17*), and during a pandemic, emotion and greed may affect not only the population but also scientific authorities and hospital workers. For example, as in the situation described by London, during the SARS epidemic, many heroic deeds were performed by scientists and health care workers, especially when SARS was an unknown microbiological enemy (*18**,**19*). Devotion to professional duty resulted in a high level of camaraderie, cohesion, and encouragement in hospitals in Asia (*18*), as among the plague survivors in London’s novel. However, the haunting fear of acquiring and spreading the disease to families, friends, and colleagues may also lead to understandable selfishness and cowardice in health providers (*20*). During the SARS crisis, for example, some physicians and nurses in Asia resigned, realizing that the profession was not for them (*18*). 

Finally, London’s work inspires reflection on the role of media during pandemics. In London’s novel, newspapers, wires, and phone calls were the only tools for obtaining information on epidemic spread: “The man who sent this news, the wireless operator, was alone with his instrument on the top of a lofty building. […] He was a hero, that man who stayed by his post—an obscure newspaperman, most likely” (*1*). Today, the main sources of information on pandemics are widely available and include the mass media, such as television, radio, and print media such as magazines and newspapers; the Internet appears to be only partly used and mainly limited to younger age groups (*21*). In London’s novel, the role of media seems to be positive (the “newspaperman” was looked upon as a hero as well as bacteriologists), but in modern times, the media are generally accused of exaggerating the risks of an epidemic and contributing to public misunderstandings of public health research evidence. Media reporting can sometimes appear to lower trust in scientific evidence, guiding public fear and spreading widely and almost instantaneously false information and exaggerated panic in public opinion (*22*). During the SARS outbreak, for example, propagation of redundant information and panic prompted reactions that were out of proportion to the risk posed by the disease (*23*). Media coverage can directly affect public risk perceptions, and recent studies have shown that media-triggered public concern may affect health-related personal measures taken during pandemics (*24**,**25*). International scientific literature has shown that, in more recent epidemics, media coverage may have had a positive influence on disease perception (*26**,**27*) and, in particular, on vaccination campaigns (*28**,**29*). As in London’s novel, the media may be a useful resource in controlling epidemic fear, enabling a bridge to be created between government/science and public opinion (*30*). 

Even though it was published a century ago, The Scarlet Plague presents the same concerns we face today, as demonstrated by the subsequent great success of this novel and the continuing literary topos of plague. Indeed, in the following decades, London’s novel inspired other literary works, including Earth Abides by George R. Stewart in 1949, I Am Legend by Richard Matheson in 1954, and The Stand by Stephen King in 1978, as well as modern blockbuster movie such as 12 Monkeys (1995), 28 Days Later (2002), Carriers (2009), and Contagion (2011).
